# MicroRNAS in endometrial cancer: recent advances and potential clinical applications

**DOI:** 10.17179/excli2014-590

**Published:** 2015-02-02

**Authors:** Megumi Yanokura, Kouji Banno, Miho Iida, Haruko Irie, Kiyoko Umene, Kenta Masuda, Yusuke Kobayashi, Eiichiro Tominaga, Daisuke Aoki

**Affiliations:** 1 Department of Obstetrics and Gynecology, Keio University School of Medicine, Tokyo, Japan

**Keywords:** microRNA, endometrial cancer, biomarker, OncomiR, tumor suppressor miR

## Abstract

Endometrial cancer is a common malignant gynecological tumor, but there are few biomarkers that are useful for early and accurate diagnosis and few treatments other than surgery. However, use of microRNAs (miRNAs) that induces gene downregulation in cells may permit effective and minimally invasive diagnosis and treatment. In endometrial cancer cells, expression levels of miRNAs including miR-185, miR-210 and miR-423 are upregulated and those of miR-let7e, miR-30c and miR-221 are downregulated compared to normal tissues, and these miRNAs are involved in carcinogenesis, invasion and metastasis. miRNAs with expression changes such as miR-181b, miR-324-3p and miR-518b may be used as prognostic biomarkers and transfection of miR-152 may inhibit cancer growth. However, most current studies of miRNAs are at a basic level and further work is needed to establish clinical applications targeting miRNAs.

## Introduction

microRNA (miRNA) is of interest as a regulatory mechanism in genomic expression. The human genome project identified approximately 20,000 genes in humans, but only 2 % of these genes are translated into proteins (International Human Genome Sequencing Consortium, 2004[24]). A large-scale transcriptome analysis showed that RNAs are also transcribed in several noncoding regions, in which gene sequences of proteins are not encoded. These so-called non-coding RNAs (ncRNAs) have complex functions including regulation of chromatin structure, splicing, and protein silencing.

miRNA is the most important class of ncRNAs. miRNAs are small RNA molecules of about 22 nucleotides that induce gene silencing. About 2,000 miRNAs have been found in humans and these molecules are important for biological activities including development; cell differentiation, proliferation and death; and metabolism (Stefani and Slack, 2008[44]; Elbashir et al., 2001[16]). Various diseases are caused by breakdown of gene regulatory networks due to changes in miRNA levels, and the relationship of miRNAs with cancer is under investigation.

One miRNA regulates many messenger RNAs (mRNAs), while cancer does not develop due to a single cause, but due to breakdown of multiple mechanisms. Therefore, in contrast to common drugs, miRNAs may be used as treatment through comprehensive regulation of expression of molecules related to cancer. Use of miRNAs may also contribute to elucidation of oncogenic and homeostatic mechanisms. In this article, we summarize the latest studies on application of miRNAs in diagnosis and treatment for endometrial cancer, based on malignant transformation associated with abnormal miRNA expression.

## miRNA and cancer

**Figure 1:**
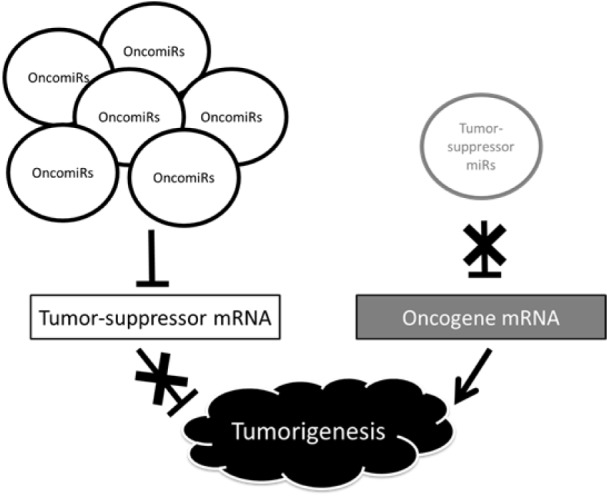
Changes in miRNA expression and oncogenic mechanisms. Upregulation of oncomiRs inhibits expression of tumor-suppressor mRNAs, resulting in enhanced oncogenesis because tumor suppression effects are lost. In contrast, downregulation of tumor-suppressor miRs reduces inhibition of expression of oncogene mRNAs, and the resultant upregulated expression of these mRNAs facilitates oncogenesis.

miRNA-induced RNA silencing is involved in homeostatic maintenance of organisms and breakdown of this mechanism causes disease. Cancer is among the best-studied diseases caused by failure of miRNA regulation. miRNAs are implicated in cancer development and progression, and expression patterns of miRNAs in normal tissues differ from those of cancer tissues. miRNAs involved in cancer are classified into oncogenic miRNAs (oncomiRs), which are upregulated in tumor tissues; and tumor suppressor miRNAs (tumor suppressor miRs), which are downregulated in tumor tissues and inhibit cancer. OncomiRs target mRNAs that suppress tumor growth, whereas tumor suppressor miRs target mRNAs that promote tumor growth. Failed regulation of both types of miRNAs promotes oncogenesis, proliferation and invasion of cancer cells, and the epithelial-mesenchymal transition (EMT) (Figure 1(Fig. 1)).

Involvement of miRNAs in human cancer was first shown in B cell chronic lymphocytic leukemia (CLL), in which expression of miR-15 or miR-16 was found to decrease (Calin et al., 2002[6]). In cancers, miR-17-92 is upregulated in lung cancer cells (Hayashida et al., 2005[20]). A large-scale analysis of miRNA expression in 540 patients with lung, breast, gastric, prostate, colon and pancreatic cancer showed that expression profiles of miRNAs in cancer tissues differ from those in normal tissues (Volinia et al., 2006[53]). miR-21 was particularly upregulated in all types of cancer, indicating that it is likely to be involved in cancer onset. Subsequently, miR-21 was shown to regulate transcriptional repressors including NFIB (Fujita et al., 2008[17]). Upregulation of miR-21 downregulates transcriptional repressors, resulting in transcriptional activation; therefore, miR-21 is considered to be a typical oncomiR.

miRNAs that are downregulated in cancer have also been found. For example, let-7, which was found in C. elegans and is involved in cell growth, is a typical miRNA precursor that is downregulated in breast, lung and gastric cancer. let-7 targets oncogenes including RAS and high mobility group AT-hook 2 (HMGA2) and has been shown to be a tumor suppressor miR (Johnson et al., 2005[27]; Lee and Dutta, 2007[33]; Yu et al., 2007[58]).

 Some miRNAs have carcinoma-specific differential expression. The relationship of many of these miRNAs with their target genes is unknown, but miR-19 is necessary for inhibition of apoptosis and oncogenesis in lymphocytes (Olive et al., 2009[39]). Chen et al. (2007[8]) found that undifferentiated cell clusters including embryonic stem (ES) cells, embryoid bodies and mouse 11-day embryos had expression profiles of miRNAs that were simpler than those of mature somatic cells. These results suggest that the differentiation stage may be defined by profiles of specific miRNAs. It is also of note that miRNA profiles in undifferentiated stem cells are similar to those of cancer cells.

## Endometrial cancer and miRNA

Endometrial cancer is the most frequent among malignant female genital cancers and approximately 80 % of cases have a histological type of endometrioid adenocarcinoma (Amant et al., 2005[1]). The oncogenic pattern of endometrial cancer and factors for metastasis and invasion have been established and the expression pattern of miRNAs differs from that of normal endometrium (Table 1(Tab. 1)) (References in Table 1: Boren et al., 2008[4]; Wu et al., 2009[54]; Chung et al., 2009[11]; Chung et al., 2012[12]; Mozos et al., 2014[38]; Dai et al., 2013[13]; Hiroki et al., 2012[21]; Tsuruta et al., 2011[51]; Banno et al., 2012[2]; Xie et al., 2011[56]; Zhou et al., 2012[61]; Kong et al., 2014[28]; Huang et al., 2014[22]; Dong et al., 2014[15]; Liu et al., 2013[35]). Among endometrial cancers, endometrioid carcinoma and papillary carcinoma also have different miRNA expression (Chan et al., 2011[7]).

In endometrial cancer, miRNAs including miR-185, miR-106a, miR-181a, miR-210, miR-423, miR-103, miR-107, miR-let7c, miR-205, miR-449 and miR-429 are upregulated and involved in oncogenesis, invasion and metastasis (Boren et al., 2008[4]; Wu et al., 2009[54]; Chung et al., 2009[11]). miR-7 is also upregulated in endometrial cancer, and invasion and cancer cell migration are inhibited by downregulation of miR-7 using an anti-miRNA antibody (Chung et al., 2012[12]). In endometrioid adenocarcinoma, upregulation of miR-27 expression is surgical stage-dependent. miR-27 contributes to survival of tumor cells through the resultant reduced expression of FOXO1, a target gene of miR-27 that inhibits apoptosis (Mozos et al., 2014[38]).

Dai et al. (2013[13]) found that miR-200b overexpression in adenocarcinoma cell lines inhibited expression of tissue inhibitor of metalloproteinase-2 (TIMP2) and increased the level of matrix metalloproteinase (MMP) 2, indicating that miR-200b is involved in metastasis of endometrial cancer. Six miRNAs show specific expression patterns in adenocarcinoma, including miR-34b, which is methylated in the promoter region in adenocarcinoma and is involved in proliferation and invasion (Hiroki et al., 2012[21]). In contrast, multiple miRNAs including miR-let7e, miR-30c, miR-221, miR-152, miR-193, miR-204, miR-99b and miR-193b are downregulated in endometrial cancer (Boren et al., 2008[4]; Wu et al., 2009[54]; Chung et al., 2009[11]). These miRNAs inhibit oncogenesis, invasion and metastasis, and these phenomena are induced by downregulation of the miRNAs.

miRNAs are also involved in DNA methylation during progression of endometrial cancer. For example, if expression of miR-129-2 is enhanced by epigenetic mechanisms including DNA demethylation and histone acetylation, expression of SRY-related high-mobility group box 4 (SOX4) is suppressed and growth of endometrial cancer is inhibited. In endometrial cancer, this mechanism fails and SOX4 is overexpressed (Huang et al., 2009[23]). miR-129-2 is involved in microsatellite instability and methylation of human mutL homolog 1 (hMLH1), a DNA mismatch repair (MMR) gene implicated in progression of type I endometrial cancer. hMLH1 is frequently methylated in endometrial cancer tissues and may induce mutation of cancer-associated genes including human mutS homolog 6 (hMSH6), type II transforming growth factor-beta (TGF-βII), Bcl2-associated X protein (BAX) and PTEN (Banno et al., 2012[2]). In addition to miR-129-2, miR-203 also regulates SOX4, and methylation of these miRNAs may lead to development of endometrial cancer (Huang et al., 2014[22]).

Expression of miR-152 is regulated by methylation and is downregulated in endometrial cancer. miR-152 inhibits expression of targets including DNA methyltransferase (DNMT1), E2F3, met proto-oncogene (MET) and rapamycin-insensitive companion of mTOR (Rictor), all of which are implicated in oncogenesis. Thus. treatment targeting miR-152 and use of this miRNA as a biomarker have potential (Banno et al., 2012[2]; Tsuruta et al., 2011[51]). miR-106b inhibits EMT and TWIST1 (Dong et al., 2014[15]) and metastasis is promoted by downregulation of miR-106b. let-7a inhibits Aurora-B and reduces the level of Aurora-B protein, with resultant inhibition of endometrial cancer onset (Liu et al., 2013[35]). miR-204 also regulates metastasis and invasion of tumor cells in the endometrium. A target of miR-204, forkhead box C1 (FOXC1), regulates metastasis and invasion in human endometrial cancer-derived HEC1A cells. In endometrial cancer, FOXC1 is overexpressed due to downregulation of miR-204 and this promotes metastasis and invasion of tumor cells (Chung et al., 2012[12]).

miR-30c acts directly on metastasis-associated gene-1 (MTA1). miR-30c inhibits cell proliferation in the endometrium via regulation of MTA1, and thus downregulation of miR-30c may be involved in typeⅠandⅡendometrial cancer (Xie et al., 2011[56]; Zhou et al., 2012[61]). miR-30c is also downregulated by estradiol (E2) in estrogen receptor (ER)-positive Ishikawa cells and ER-negative HEC1B cells, which indicates that estrogen regulates miR-30c in the endometrium and is involved in oncogenesis of endometrial cancer (Kong et al., 2014[28]). Lin et al. (2008[34]) showed that miR-302 induces demethylation of overall genomic DNA, and consequently activates transcription factors including Oct4, Sox2, Nanog and Lin28. Expression of specific genes in human ES cells causes reprogramming in these cells, with somatic cells changed to pluripotent stem (mirPS) cells. miR-302 inhibits tumorigenesis in various cancers, including through direct inhibition of cyclin D1 and indirect inhibition of CDK1 (Yan et al., 2014[57]).

Many important findings have been described in this section, but it is also clear that an understanding of the mechanisms of miRNAs in endometrial cancer requires further studies.

## Application of miRNA in endometrial cancer as a biomarker

Valadi et al. reported the initial finding of extracellular miRNAs in exosomes (Valadi et al., 2007[52]). Exosomes are extracellular vesicles and miRNA carriers that are released from cells into blood. Catabolic enzymes for miRNA are present in serum and it was originally thought that miRNAs could not exist in blood. However, miRNAs in exosomes are stable and are present in blood (Chim et al., 2008[10]; Gilad et al., 2008[18]). Exosomes released from cancer cells may have a relationship with mechanisms including immunosuppression, drug resistance and angiogenesis. Thus, miRNAs in exosomes reflect cell characteristics and overexpressed miRNAs in cancer cells are included in exosomes released from these cells. Lawrie et al. (2008[31]) first showed that cancer-specific miRNAs were effective biomarkers, and subsequently development of biomarkers using miRNAs has increased.

Several miRNAs have specific expression profiles in particular carcinomas and are likely to be useful as biomarkers. Analysis of miRNAs may classify cancer more correctly than analysis of 20,000 mRNAs (Lu et al., 2005[36]). Lung cancer with downregulation of let-7 has a poor prognosis after surgery, indicating that miRNAs may be markers for prognosis, as well as for early diagnosis (Takamizawa et al., 2004[45]). Most patients with endometrial cancer are diagnosed at an early clinical stage. However, the 5-year survival rate of patients in whom endometrial cancer is detected at an advanced stage is low, ranging from 10 % to 29 %. Therefore, development of a specific marker is required for early detection of endometrioid adenocarcinoma (Bansal et al., 2009[3]). For example, Tan et al. (2010[47]) found that upregulation of miR-155 was related to cancer stage and lymph node metastases in a study of miR-155 in serum that had the goal of detection of differences in miRNA expression among endometrioid adenocarcinoma, normal endometrium, and other tissue types. 

Endometriosis is one of precancerous lesion of endometrial cancer that is a target for early diagnosis. A combination of miR-199a and miR-542-3p can be used to diagnose endometriosis with a sensitivity of 96.61 % and specificity of 79.66 %, and this approach may serve as a noninvasive marker in practice (Yu et al., 2012[59]). Torres et al. (2012[48]) found that expression of miR-99a, miR-100 and miR-199b was upregulated in serum of patients with endometrioid adenocarcinoma, and showed that a combination of miR-99a and miR-199b had a higher diagnostic value than each miRNA alone. Analysis of miRNAs in serum in a genome-wide study showed that a combination of four serum miRNAs, miR-222, miR-223, miR-186 and miR-204, can be used to diagnose endometrioid adenocarcinoma with high probability. Use of this combination for diagnosis gave an area under the ROC curve of 0.927, which was higher than that for the currently used marker, CA-125 (0.673).

TypeⅡ endometrial cancer is difficult to diagnose because it involves de novo oncogenesis and has no precancerous lesion. However, several miRNAs including miR-125b may be useful for diagnosis of endometrial cancer. Expression of miR-125b in typeⅡendometrial cancer cells is significantly upregulated compared to that in typeⅠ cells. Tumor protein p53 inducible nuclear protein 1 (TP53INP1) gene, a target of miR-125b, may be related to malignancy of typeⅡendometrial cancer because cancer cells proliferate when this gene is not regulated (Jiang et al., 2011[25]). V-erb-b2 erythroblastic leukemia viral oncogene homolog 2 (ERBB2), a second target of miR-125b, is also involved in invasion of cancer cells (Shang et al., 2012[42]). miR-194 may also be a biomarker for prognostic diagnosis of endometrial cancer. miR-194 is markedly decreased in endometrial cancer and this decrease is correlated with the stage of cancer and is associated with prognosis (Zhai et al., 2013[60]). Administration of pre-miRNA showed that miR-194 targets the oncogene BMI1 and inhibits the EMT phenotype and EC cell invasion (Dong et al., 2011[14]).

KRAS mutation frequently occurs in typeⅡ endometrial cancer. Expression levels of miR-181b, miR-324-3p and miR-518b are downregulated in cancer with a KRAS mutation (Lee et al., 2014[32]). Thus, miR-181b, miR-324-3p and miR-518b may be prognostic markers. miR-205 targeting PTEN is also associated with decreased survival. Expression profiles of miRNAs in endometrioid adenocarcinoma may also be implicated in clinicopathologic characteristics (Torres et al., 2013[49]; Tsukamoto et al., 2014[50]), with expression of several miRNAs showing a relationship with International Federation of Gynaecology and Obstetrics (FIGO) staging, cancer grade, recurrence and lymph node metastasis.

## Application of miRNA for treatment of endometrial cancer

miRNAs have a close relationship with cancer, and therefore may be useful for cancer treatment. Two approaches have been proposed as therapeutic strategies involving miRNAs. The first is replacement therapy with a particular miRNA for which downregulation is associated with disease onset. The second is replacement of molecules inhibiting a certain miRNA for which upregulation is associated with onset. The first approach includes treatment with viral vectors and ribosomes. miR-16 supplemented in bone metastasis model mice with prostate cancer reduced proliferation of local tumors and bone metastasis was significantly inhibited without adverse reactions. These results suggest that supplementation of downregulated miRNA can have a therapeutic effect (Takeshita et al., 2010[46]). Similarly, administration of miR-143 inhibited metastasis in pulmonary metastasis model mice with osteosarcoma (Osaki et al., 2011[40]). The second approach includes inhibition of a target miRNA using locked nucleic acid (LNA). Using this approach, activities of miR-122 in primates with hepatitis C were inhibited without serious adverse reactions and HCV proliferation was also inhibited (Randall et al., 2007[41]; Lanford et al., 2010[30]).

Studies of miRNAs have revealed detailed mechanisms of drugs. Such drugs include bortezomib, an inhibitor of ubiquitin-dependent proteolysis that kills tumor cells in vitro and in animal models. This drug blocks proteasome activity, arrests cells in G2/M phase, induces apoptosis, and inhibits development of endometrial cancer. The action of miR-17-5p on p21 increased the efficacy of bortezomib (Shen et al., 2013[43]).

Drug sensitivity of cancer can also be influenced using miRNAs. In endometrial cancer, expression of miR-34c, which regulates metastasis, cell death and invasion, is markedly downregulated, and a combination of a miR-34c mimic with cisplatin improved the drug efficacy in cell lines (Jiang et al., 2013[26]). Expression of miR-200b, miR-200c and miR-429, which inhibit transcription factor AP-2α, is upregulated in endometrial cancer and the rate of expression is positively correlated with resistance to cisplatin. However, SNP (rs1045385A>C) decreases the effect on AP-2α, and sensitivity to cisplatin is increased (Wu et al., 2011[55]). These studies suggest that a comprehensive investigation of which miRNA causes resistance or increases sensitivity to a particular drug will improve selection of drug treatment.

miRNAs themselves can be used for cancer treatment as a means of increasing expression of tumor suppressor genes and inhibiting oncogenes. Administration of a tumor suppressor miR, miR-152, in vitro and in vivo gave significant tumor suppression (Tsuruta et al., 2011[51]). However, no miRNA is currently used in clinical practice, in part because application of miRNAs depends on effective and cancer cell-specific transport. miRNAs exist stably in various tissues and structures, and therefore should be relatively easy to transport into cells via blood vessels and other structures (Mitchell et al., 2008[37]). Transport methods for short interfering RNA (siRNA) should also be applicable to miRNA (Gondi and Rao, 2009[19]; Broderick and Zamore, 2011[5]) due to the structural similarity of miRNA and siRNA. Nanoparticles contribute to the stability of miRNAs (Kong et al., 2012[29]) and liposome-polycation-hyaluronic acid (LPH) modified with a single-chain antibody fragment (scFv) has been used for transport of miRNAs and siRNAs into cancer cells, with resultant downregulation of target genes (c-Myc, MDM2 and VEGF). Intravenous injection of nanoparticles modified with a humanized monoclonal antibody GC4 and miR-34a and two siRNAs also resulted in significant accumulation in cancer cells and inhibition of expression of target genes (Chen et al., 2010[9]).

These results suggest that miRNAs can be transported specifically into cancer cells using nanoparticles modified with antibodies to cancer cell surface markers, and that a combination of siRNAs and miRNAs can increase the efficiency of gene regulation. Effects of miRNAs are relatively mild in comparison with molecular targeted drugs, but miRNAs inhibit expression of multiple target molecules and cause fewer adverse reactions in normal cells.

## Conclusion

It is only 20 years since miRNAs were discovered, but many mechanisms have now been elucidated. Cancer cells include miRNAs with cancer-specific upregulation and downregulation, and these miRNAs are involved in oncogenesis, invasion and metastasis. miRNAs with expression patterns that differ from those in normal tissues, such as miR-125b, may be useful in treatment and as biomarkers for endometrial cancer. These miRNAs are currently being studied at an investigational level prior to development of clinical applications.

## Acknowledgements

We thank Dr. Y. Matsubara and Dr. E. Yuasa for helpful assistance. The authors gratefully acknowledge grant support from the Japan Society for the Promotion of Science (JSPS) through a Grant-in-Aid for Scientific Research (KAKENHI), a Grant-in-Aid for Scientific Research (C) (25462608), and a Grant-in-Aid for Young Scientists (B) (24791718); the Medical Research Encouragement Prize of The Japan Medical Association; and the Keio Gijyuku Academic Development Fund. The funders had no role in study design, data collection and analysis, decision to publish, or preparation of the manuscript.

## Conflict of interest

The authors declare that they have no conflict of interest.

## Figures and Tables

**Table 1:**
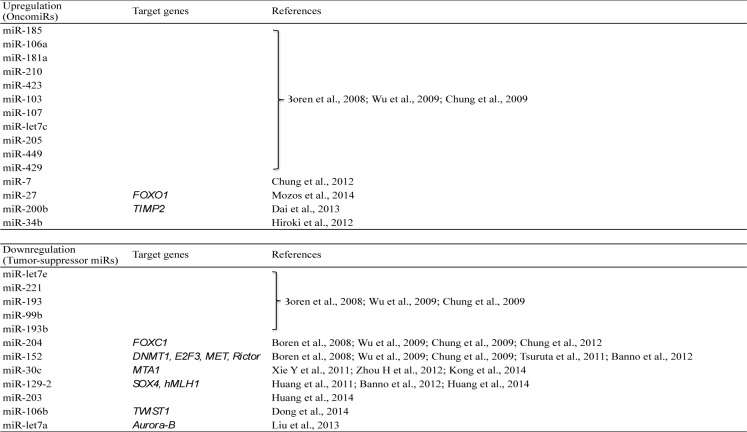
Changes in expression of miRNAs in endometrial cancer
